# Advances in Engineering Nucleotide Sugar Metabolism for Natural Product Glycosylation in *Saccharomyces cerevisiae*

**DOI:** 10.1021/acssynbio.3c00737

**Published:** 2024-05-31

**Authors:** Samantha A. Crowe, Yuzhong Liu, Xixi Zhao, Henrik V. Scheller, Jay D. Keasling

**Affiliations:** † Department of Chemical & Biomolecular Engineering, University of California, Berkeley, California 94720, United States; ‡ California Institute of Quantitative Biosciences (QB3), University of California, Berkeley, California 94720, United States; § Joint BioEnergy Institute, Emeryville, California 94608, United States; ∥ Environmental Genomics and Systems Biology Division, Lawrence Berkeley National Laboratory, Berkeley, California 94720, United States; ⊥ Department of Plant and Microbial Biology, University of California, Berkeley, California 94720, United States; # Department of Bioengineering, University of California, Berkeley, California 94720, United States; ∇ Division of Biological Systems and Engineering, Lawrence Berkeley National Laboratory, Berkeley, California 94720, United States; ○ Center for Biosustainability, Technical University of Denmark, 2800 Kongens Lyngby, Denmark; ◆ Center for Synthetic Biochemistry, Shenzhen Institute of Advanced Technology, Chinese Academy of Sciences, Shenzhen 518055, China

**Keywords:** Uridine diphosphate sugar metabolism, nucleotide sugar, *Saccharomyces cerevisiae*, glycosylation, natural products, glycosides

## Abstract

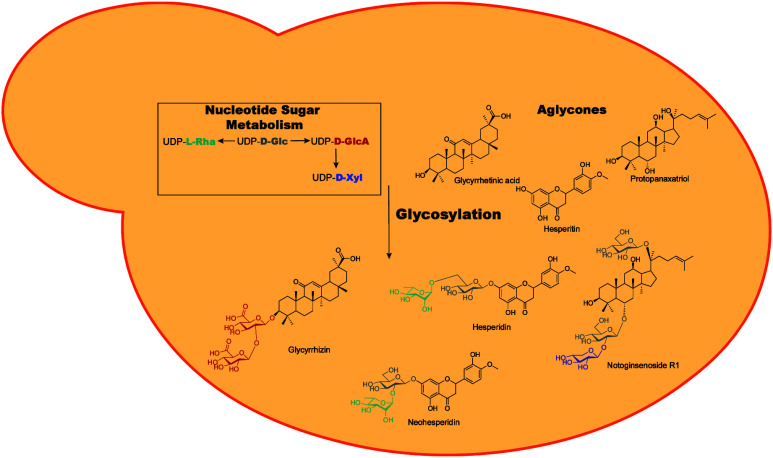

Glycosylation is a ubiquitous modification present across all of biology, affecting many things such as physicochemical properties, cellular recognition, subcellular localization, and immunogenicity. Nucleotide sugars are important precursors needed to study glycosylation and produce glycosylated products. *Saccharomyces cerevisiae* is a potentially powerful platform for producing glycosylated biomolecules, but it lacks nucleotide sugar diversity. Nucleotide sugar metabolism is complex, and understanding how to engineer it will be necessary to both access and study heterologous glycosylations found across biology. This review overviews the potential challenges with engineering nucleotide sugar metabolism in yeast from the salvage pathways that convert free sugars to their associated UDP-sugars to *de novo* synthesis where nucleotide sugars are interconverted through a complex metabolic network with governing feedback mechanisms. Finally, recent examples of engineering complex glycosylation of small molecules in *S. cerevisiae* are explored and assessed.

## Introduction

**Figure 1:**
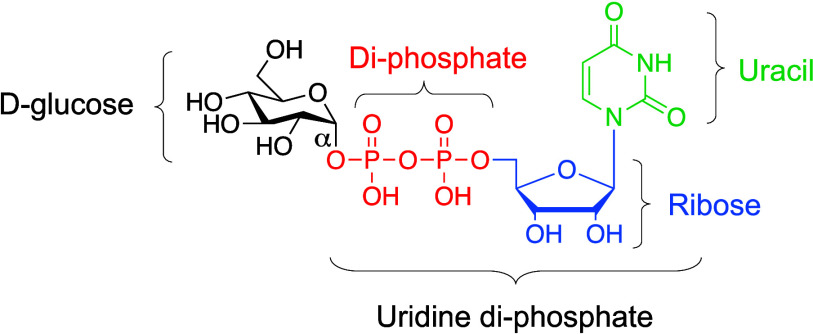
Structure of uridine diphosphate α-d-glucose (UDP-d-Glc) consisting of uracil, ribose, and diphosphate (which compose UDP) and d-glucose. This is a common activated form of d-glucose which is essential for recognition by UDP-dependent glycosyltransferases and its use in glycosylation.

Glycosylation is an important modification found across biology present in proteins, lipids, and natural products, and it affects structure and function as well as stability and solubility.^1−3^ It is also vital for energy storage as well as cellular communication. Glycosylation is achieved in biology through glycosyltransferases, with the largest family being uridine-diphosphate-dependent glycosyltransferases (UGTs).^1^ Nucleotide diphosphate (NDP)-sugars serve as the monomer substrates for more than 90% of glycosylation reactions and are essential building blocks for naturally occurring polysaccharides and glycoconjugates.^1^ The most common glycosylation precursor is uridine diphosphate α-d-glucose (UDP-d-Glc) (Figure 1). Although there are a wide variety of different nucleotide sugars, glycosylation reactions involving these precursors are understudied due to lack of availability of nucleotide sugars.^4^

*Saccharomyces cerevisiae* is a powerful platform utilized to both elucidate biosynthetic pathways and aid in the scale-up of the production of many different types of natural products.^5−10^ Yeast is also more likely to be easier to engineer to make complex molecules compared with other hosts. Unlike prokaryotic hosts, yeast contains an endoplasmic reticulum (ER) necessary for membrane-bound enzymes involved in plant natural product biosynthesis. Yeast does have an abundant amount of UDP-d-Glc that has been used to glycosylate different molecules such as ginsenosides^11^ and steviol.^12^ However, there are only a handful of examples of yeast producing a molecule not glycosylated with glucose.^8,9,13−15^ This is vastly limiting, considering the plethora of different sugars synthesized across biology that serve as building blocks of a wide variety of different products. In order to increase the diversity of products, the diversity of nucleotide sugars accessible in yeast must increase.

**Figure 2:**
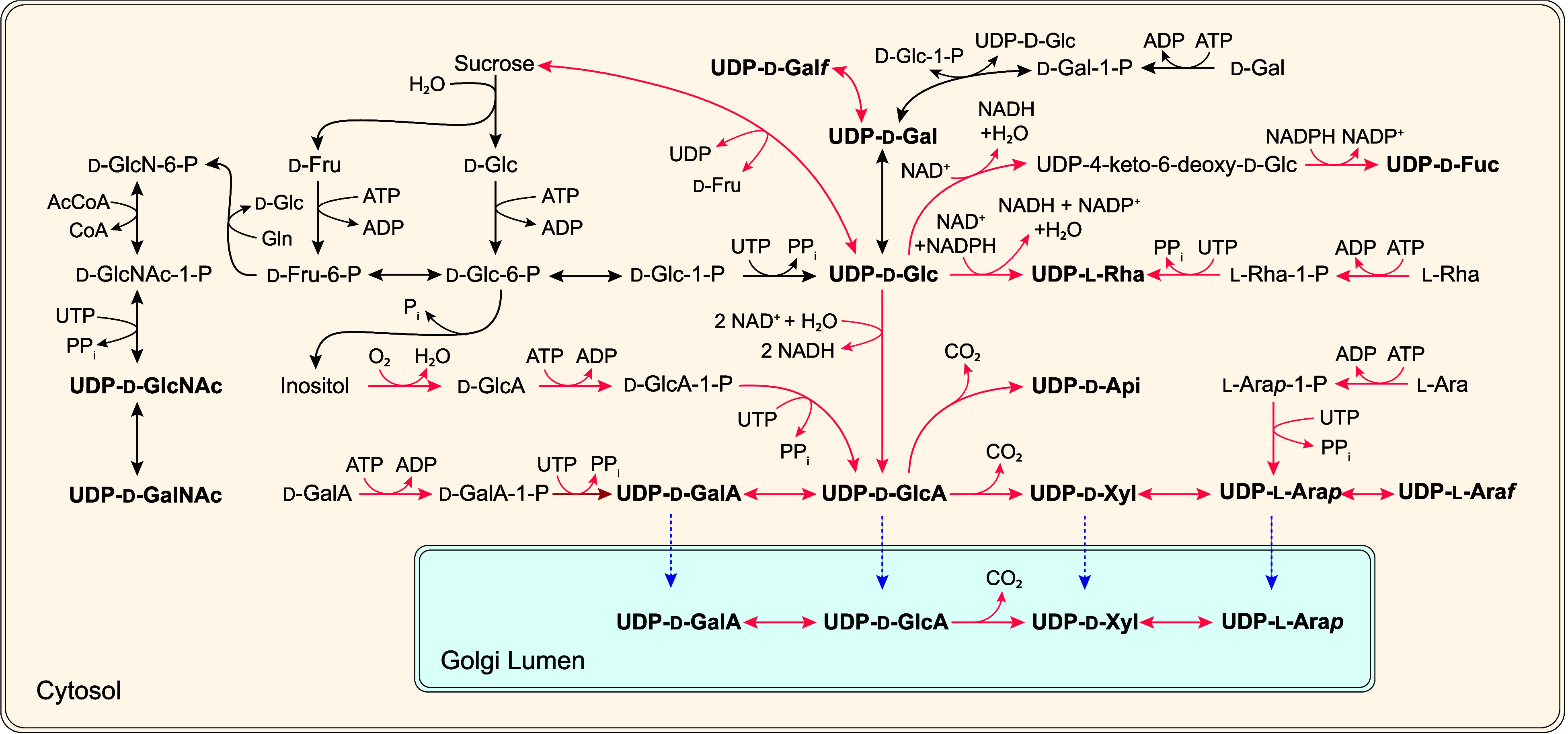
Major pathways to produce different UDP-sugars from simple sugars *in vivo*. Pathways that start from individual sugars with known enzymes are included. Gray dashed arrows indicate transporters from the cytosol into the lumen of the Golgi apparatus. Many UDP-sugars are transported into the Golgi apparatus by specific UDP-sugar transporters and can be interconverted in the Golgi lumen, but only a few are shown as examples. Enzymes in the cytosol that interconvert or salvage UDP-sugars can be found either purely cytosolic or anchored to the cell membrane, Golgi apparatus, *etc.S. cerevisiae*’s native metabolism is depicted with black arrows, and non-native pathways to access other UDP-sugars are depicted with red arrows. A full list of enzymes needed for each part of this metabolic network can be found in Table S1.

Nucleotide sugars can be made in many different ways (Figure 2).^16^ Free sugars, such as rhamnose and arabinose, can be salvaged and converted to their activated nucleotide forms.^16^ Alternatively, nucleotide sugars can be accessed by enzymes that provide a biosynthetic network to interconvert existing nucleotide sugars. This process allows for broad structural variety of NDP-sugar monomers *via* reactions such as oxidation, decarboxylation, epimerization, *etc.*(16) Nucleotide sugar metabolism primarily takes place in the cytosol, though it can also occur in the Golgi apparatus, where polysaccharide biosynthesis and protein glycosylation occur. This metabolism is complex and complicated with different feedback mechanisms that make it difficult to tune nucleotide sugar conversion.^16,17^ This review will focus on the biosynthesis of UDP-sugars (see Table 1) and potential challenges with engineering their metabolism as well as recent engineering efforts toward producing complex glycosylated products in yeast.

**Table 1:** Sugars Discussed in This Review and Their Activated Forms

sugar	activated form	abbreviated name
d-glucose	UDP-α-d-glucose	UDP-d-Glc
d-galactose	UDP-α-d-galactose	UDP-d-Gal
d-galactofuranose	UDP-α-d-galactofuranose	UDP-d-Gal*f*
d-glucuronic acid	UDP-α-d-glucuronic acid	UDP-d-GlcA
d-galacturonic acid	UDP-α-d-galacturonic acid	UDP-d-GalA
*N*-acetyl-d-glucosamine	UDP-α-*N*-acetyl-d-glucosamine	UDP-d-GlcNAc
*N*-acetyl-d-galactosamine	UDP-α-*N*-acetyl-d-galactosamine	UDP-d-GalNAc
d-apiose	UDP-α-d-apiose	UDP-d-Api
d-xylose	UDP-α-d-xylose	UDP-d-Xyl
l-arabinopyranose	UDP-β-l-arabinopyranose	UDP-l-Ara*p*
l-arabinofuranose	UDP-β-l-arabinofuranose	UDP-l-Ara*f*
l-rhamnose	UDP-β-l-rhamnose	UDP-l-Rha
d-fucose	UDP-α-d-fucose	UDP-d-Fuc

## Expanding Nucleotide Sugar Metabolism in Yeast

For practicality, it is easiest to make heterologous UDP-sugars in yeast by using either the salvage pathway or the *de novo* pathway.

### Salvage Pathway

**Figure 3:**
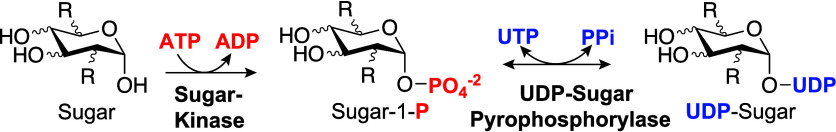
Salvage pathway. The salvage pathway consists of the phosphorylation of a sugar by an associated kinase and then the transfer of a UMP moiety from UTP by a UDP-sugar pyrophosphorylase.

The salvage pathway involves converting a simple sugar to its UDP-sugar form by a specific kinase and a UDP-sugar pyrophosphorylase that can be either specific or promiscuous. The kinase phosphorylates the sugar at the C1 residue to make the sugar-1-phosphate, and then the UDP-sugar pyrophosphorylase converts it to the UDP-sugar (see Figure 3). Kinases are typically highly specific for their sugar substrates.^16^ For some sugars, such as d-xylose and d-fucose, the kinases have not been described, though there have been efforts to engineer promiscuous kinases.^18^ UDP-sugar pyrophosphorylases are either specific, in the case of UDP-d-Glc pyrophosphorylases (UGPs) and UDP-d-*N*-acetylglucosamine pyrophosphorylases (UAGPs), or quite promiscuous, such as in the case of “sloppy” UDP-sugar pyrophosphorylases (USPs) that can show different levels of promiscuity toward different sugar-1-phosphates.^19^

The salvage pathway may be advantageous for the synthesis of specific UDP-sugars that have readily available substrates and pathway enzymes. One such pathway is the *myo*-inositol pathway for UDP-d-GlcA production, where *myo*-inositol is oxidatively cleaved by *myo*-inositol oxidase to form d-glucuronic acid, which can then be converted to UDP-d-GlcA *via* the salvage pathway with d-glucuronic acid kinase followed by UDP-sugar pyrophosphorylase.^20^ UDP-l-Ara*p* can also be synthesized from free arabinose utilizing the salvage pathway with arabinokinase and UDP-sugar pyrophosphorylase.^21^ The salvage pathway is also orthogonal to yeast’s native metabolism and does not rely on nucleotide sugar interconversion, which may suffer from low yield. This pathway may be the best way to synthesize nonstandard sugars such as fluorinated sugars.^22^ It is unfortunately limited in other ways, as not every sugar has a known kinase (*e.g.*, xylose) and individual sugars can be prohibitively expensive to feed in or are not commercially available. For the production of a wide variety of UDP-sugars, the salvage pathway is not very optimal.

### *De Novo* Pathway

**Figure 4:**
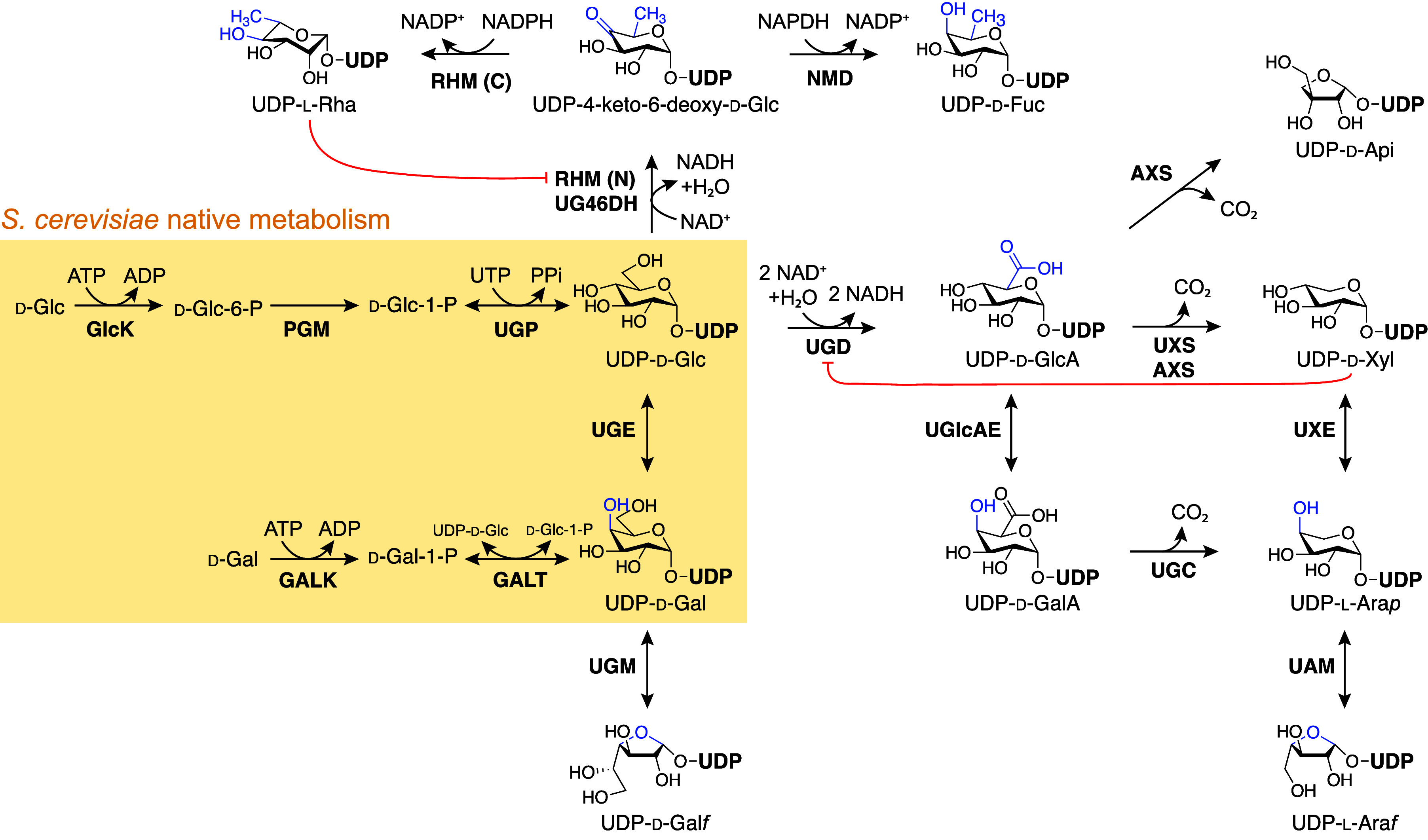
*De novo* synthesis of UDP-sugars from UDP-d-Glc/UDP-d-Gal starting with d-Gal and d-Glc feed-in. *S. cerevisiae*’s native metabolism is shaded in orange. Free glucose can be phosphorylated by glucokinase (GlcK) or hexokinase to d-Glc-6-P, which is then mutated by phosphoglucomutase (PGM) to d-Glc-1-P, which in turn is converted to UDP-d-Glc by UDP-d-Glc pyrophosphorylase (UGP). Free galactose can be phosphorylated by GALK and converted by GALT with UDP-d-Glc to form UDP-d-Gal and d-Glc-1-P. UDP-d-Gal is also interconverted with UDP-d-Glc by UDP-d-Glc 4-epimerase (UGE). UDP-l-Rha is made from UDP-d-Glc using UDP-rhamnose synthase (RHM), a three-domain enzyme composed of a 4,6-dehydratase, denoted as RHM (N), and a 3,5-epimerase and 4-keto-reductase, denoted as RHM (C). UDP-d-Fuc is made by UDP-glucose-4,6-dehydratase (UG46DH) *via* a common keto-sugar intermediate, UDP-4-keto-6-deoxy-d-Glc, which is then reduced by neomenthol dehydrogenase (NMD). UDP-l-Rha is a known inhibitor of several UG46DH domains of RHM. UDP-d-GlcA is synthesized from UDP-d-Glc by UDP-d-Glc 6-dehydrogenase (UGD). UDP-d-GlcA 4-epimerase (UglcAE) interconverts UDP-d-GlcA and UDP-d-GalA by C4 epimerization. The C6 carboxylic acid of UDP-d-GlcA is decarboxylated by UDP-d-Xyl synthase (UXS) to yield UDP-d-Xyl. UDP-d-Api/Xyl synthase (AXS) can convert UDP-d-GlcA to a mixture of UDP-d-Xyl and UDP-d-Api. UDP-l-Ara*p* and UDP-l-Ara*f* are the pyranose/furanose isomers of UDP-l-Ara. UDP-d-Xyl 4-epimerase (UXE) synthesizes UDP-l-Ara*p* from UDP-d-Xyl by C4 epimerization, and UDP-l-Ara mutase (UAM) performs a ring mutation of UDP-l-Ara*p* to form UDP-l-Ara*f*. Similarly, UDP-d-Gal mutase (UGM) interconverts UDP-d-Gal and UDP-d-Gal*fvia* ring rearrangement.

The *de novo* pathway interconverts UDP-sugars into different UDP-sugars directly, typically starting from UDP-d-Glc, and can access a wide variety of UDP-sugars. A broad class of nucleotide sugar interconversion enzymes carry out these reactions by modifying the sugar attached to UDP through dehydration, reduction, decarboxylation, epimerization, ring restructuring, *etc.*(16) As many sugars are either prohibitively expensive or unavailable commercially and some do not have a known kinase (*e.g.*, xylose), the *de novo* pathway using nucleotide sugar interconversion enzymes is often advantageous. Starting from UDP-d-Gal and UDP-d-Glc (as yeast makes both), many other UDP-sugars can be synthesized by the *de novo* pathway (Figure 4). A small number of these enzymes have been previously tested in yeast,^23,24^ but there are still many challenges to address in engineering these pathways.

Yeast natively makes UDP-d-Glc and UDP-d-Gal from either glucose or galactose.^25^d-Glucose can be phosphorylated to d-Glc-6-P and isomerized by phosphoglucomutase (PGM) to d-Glc-1-P, which is then converted to UDP-d-Glc by UGP. d-Galactose can be phosphorylated by galactokinase (GALK) to make d-Gal-1-P, which can then be converted by galactose-1-phosphate uridylyltransferase (GALT) with UDP-d-Glc to form UDP-d-Gal and d-Glc-1-P (this is also known as the Leloir pathway).^17^*S. cerevisiae* also contains the enzyme UDP-glucose 4-epimerase (UGE), which interconverts UDP-d-Glc and UDP-d-Gal by C4 epimerization.^25^

Most of these UDP-sugars originate from UDP-d-Glc. UDP-l-Rha is made from UDP-d-Glc *via* the UDP-l-Rha synthase (RHM), which is a large three-domain enzyme that performs a 4,6-dehydration (N-terminus), 3,5-epimerization, and 4-keto reduction (C-terminus).^24^ UDP-d-Fuc is made in a similar manner from UDP-d-Glc with a 4,6-dehydration performed by UDP-d-Glc 4,6-dehydratase (UG46DH) followed by a 4-keto reduction by a homologue of neomenthol dehydrogenase (NMD) without a 3,5-epimerization.^26^

UDP-d-GlcA is made from UDP-d-Glc by UDP-d-Glc 6-dehydrogenase (UGD), which oxidizes the C6 hydroxy residue on d-glucose to the carboxylic acid.^23^ UDP-d-GlcA can be reversibly interconverted to UDP-d-Gal *via* C4 epimerization by UDP-d-GlcA 4-epimerase (UGlcAE).^27^ UDP-d-Xyl synthase (UXS) performs a C6 decarboxylation on UDP-d-GlcA to make UDP-d-Xyl.^28^ A similar C6 decarboxylation performed by UDP-GalA decarboxylase (UGC) that converts UDP-d-GalA to UDP-l-Ara*p* has been reported in the pathogenic fungus *Ampullariella digitata*.^29^ However, UGC activity has not been reported from other species, and the putative enzyme has not been identified. UDP-d-GlcA can also be converted to UDP-d-Api by UDP-d-Api/Xyl synthase (AXS), which performs a C6 decarboxylation and ring contraction.^30^ AXS also makes UDP-d-Xyl by performing only the decarboxylation reduction. UDP-Xyl 4-epimerase (UXE) interconverts UDP-d-Xyl and UDP-l-Ara*p* to about equal molar concentrations.^31^ UDP-l-Ara*f*, the furanose form of UDP-l-Ara*p* and the primary form of arabinose found in plant saccharides,^21,32^ is made from UDP-l-Ara*p* by UDP-l-Ara mutase (UAM).^32^ This enzyme has also been named “reversible glycosylated polypeptide” (RGP).^32^ Similarly, UDP-d-Gal*f* is made from UDP-d-Gal by UDP-d-Gal mutase (UGM).^33^ Both UAM and UGM are ruled by thermodynamic equilibria that favor the pyranose form of the sugar over the furanose form: for UAM this equilibrium is 90:10 favoring UDP-l-Ara*p*,^32^ and for UGM this equilibrium is 11:1 favoring UDP-d-Gal.^33^

There are many common reactions involved in the interconversion of UDP-sugars including C6 oxidation, C6 decarboxylation, C4 epimerization, C4,6 dehydration, ring restructuring, *etc.* C4 epimerization is commonly used to interconvert UDP-d-Glc/UDP-d-Gal, UDP-d-GlcNAc/UDP-GalNAc, UDP-d-GlcA/UDP-d-GalA, and UDP-d-Xyl/UDP-l-Ara*p*. Some epimerases have been discovered to work on nucleotide sugars other than UDP-d-Glc/UDP-d-Gal, namely, UDP-d-GlcA/UDP-d-GalA and UDP-d-Xyl/UDP-l-Ara*p*, which is advantageous because UGlcAE and UXE are usually membrane-bound and active in the Golgi lumen.^34^ C4,6 dehydration followed by 4-keto reduction is common to both UDP-l-Rha and UDP-d-Fuc biosynthesis, and a similar 4,6-dehydration, 3,5-epimerization, and 4-keto reduction is used to convert GDP-d-mannose to GDP-l-fucose.^16^

It is widely observed across biology that UDP-d-Xyl is a strong inhibitor of its upstream enzyme UGD even in organisms that do not make UDP-d-Xyl.^23,35^ UDP-d-Xyl allosterically binds to UGD with a very high affinity. Previously, UGD1 from *Arabidopsis thaliana* was measured to have a *K*_i_ of 4.9 μM for UDP-d-Xyl and 99 μM for UDP-d-GlcA.^23^ It is also a known inhibitor of other enzymes involved in UDP-sugar metabolism such as UGP,^36^ UXS,^28^ and RHM.^24^ There have been efforts to alleviate this inhibition of UGD by implementing point mutations on the *Homo sapiens* UGD that increased the apparent *K*_i_ of UDP-d-Xyl 10-fold.^37^ UDP-d-Xyl is hypothesized to be a very important nucleotide sugar in regulating nucleotide sugar metabolism, mostly in maintaining a high UDP-d-Glc concentration. Its strong inhibition of UGD likely helps control the irreversible conversion of UDP-d-Glc and the synthesis of UDP-pentose sugars.

Another common feedback mechanism is the inhibition of the N-terminus of RHM (the 4,6-dehydratase domain) by the end product of UDP-l-Rha.^24^ This inhibition again is likely to maintain high UDP-d-Glc levels *in vivo*, as the synthesis of UDP-l-Rha from UDP-d-Glc is irreversible. As UDP-d-Xyl is also a known inhibitor of the N-terminal domain of RHM,^24^ it seems that UDP-d-Xyl is an important overall regulator of UDP-sugar metabolism. In terms of *de novo* synthesis, there seems to be either reversible interconversion or high inhibition that serves to prevent UDP-d-Glc depletion. UDP-d-Glc is an important metabolite that is used in many different pathways, *e.g.*, as an important precursor to cell wall biosynthesis, as a precursor to glycogen synthesis, as a precursor to other downstream UDP-sugars, and as a cell signaling molecule.^16,23,37−39^

## Recent Efforts in Engineering Glycosylation in *S. cerevisiae*

**Figure 5:**
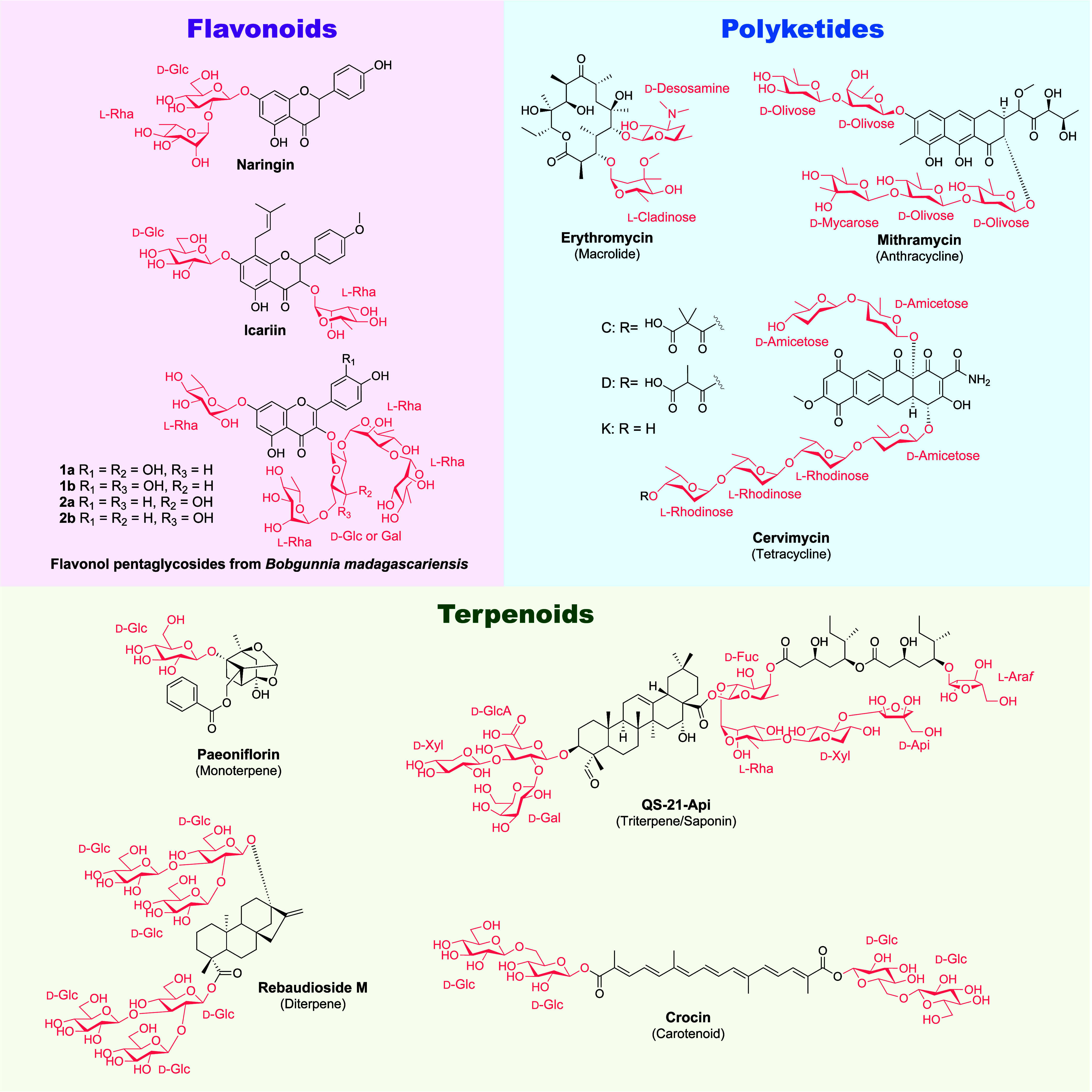
Examples of glycosylated natural products from flavonoids to polyketides and terpenoids. Sugars are labeled and highlighted in red.

Engineering *S. cerevisiae* to produce glycosylated molecules requires a variety of tools and strategies. Examples of different glycosylated natural products include flavonoids, polyketides, and terpenoids, which can be found in nature to be decorated by a variety of different sugars (Figure 5). Many of the molecules of interest for production in yeast are derived from terpene and flavonoid metabolism.

**Figure 6:**
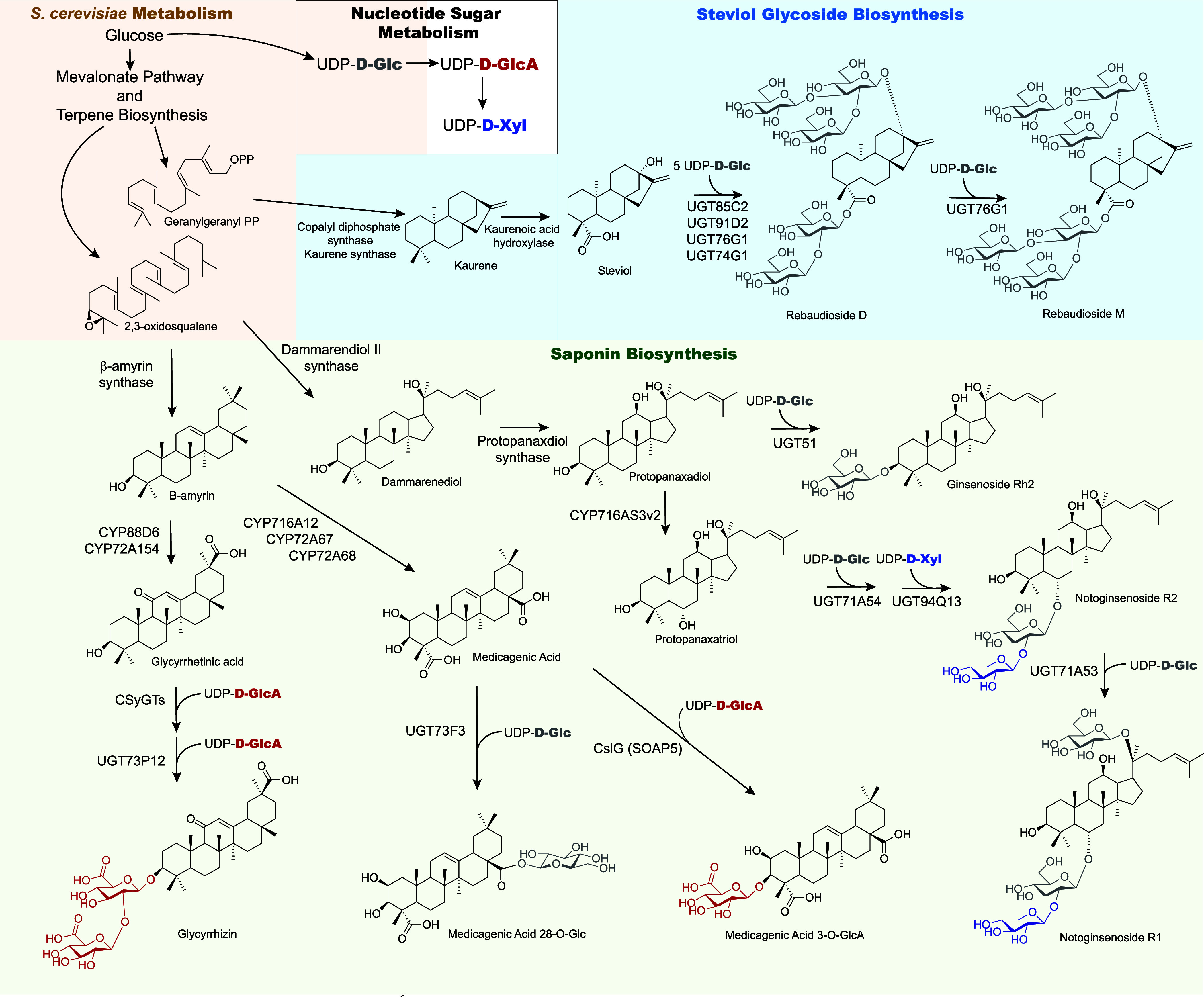
Examples of glycosylated terpene products made in *S. cerevisiae* and their biosynthetic pathways. Native yeast metabolism is shaded in orange, steviol glycoside biosynthesis in blue, and saponin biosynthesis in green.

Terpenoids are a diverse class of molecules, comprising more than 50,000 known molecules, mostly coming from plants.^40^ Examples of terpenes include saponins, which are derived from triterpene (C30) 2,3-oxidosqualene. Saponins are a structurally diverse set of natural products found in many types of plants, consisting of a triterpenoid or steroid core that is glycosylated at one or more residues.^41,42^ They are widely desired for a variety of purposes, ranging from the adjuvant and anticancer activity of QS-21^43^ to the anti-inflammatory aescin^44^ and the low-calorie sweetener glycyrrhizin, which may also be a COVID-19 therapeutic.^45,46^ The biosynthesis of saponins requires 2,3-oxidosqualene cyclases (OSCs) that cyclize 2,3-oxidosqualene (derived from the mevalonate pathway) to a four- or five-ring structure core, typically followed by cytochrome P450-dependent monooxygenases (P450s) that are localized to the ER and oxidize selected carbon residues on the 30-carbon core. Further modification involves UDP-glycosyltransferases (UGTs) that glycosylate selected oxygen residues with specific sugars and are typically localized in the cytoplasm (Figure 6). Various other enzymes, such as methyltransferases, acyltransferases, *etc.*, can also be a part of saponin synthesis.^41^ The synthesis of other terpene glycosides such as rebaudioside M, a diterpene glycoside, similarly involves P450s and UGTs.^40,47^*S. cerevisiae* natively harbors the mevalonate-based terpene synthesis pathways, so biosynthesis typically requires tailoring enzymes from plants such as P450s, UGTs, and enzymes to increase nucleotide sugar supply or diversity.

**Figure 7:**
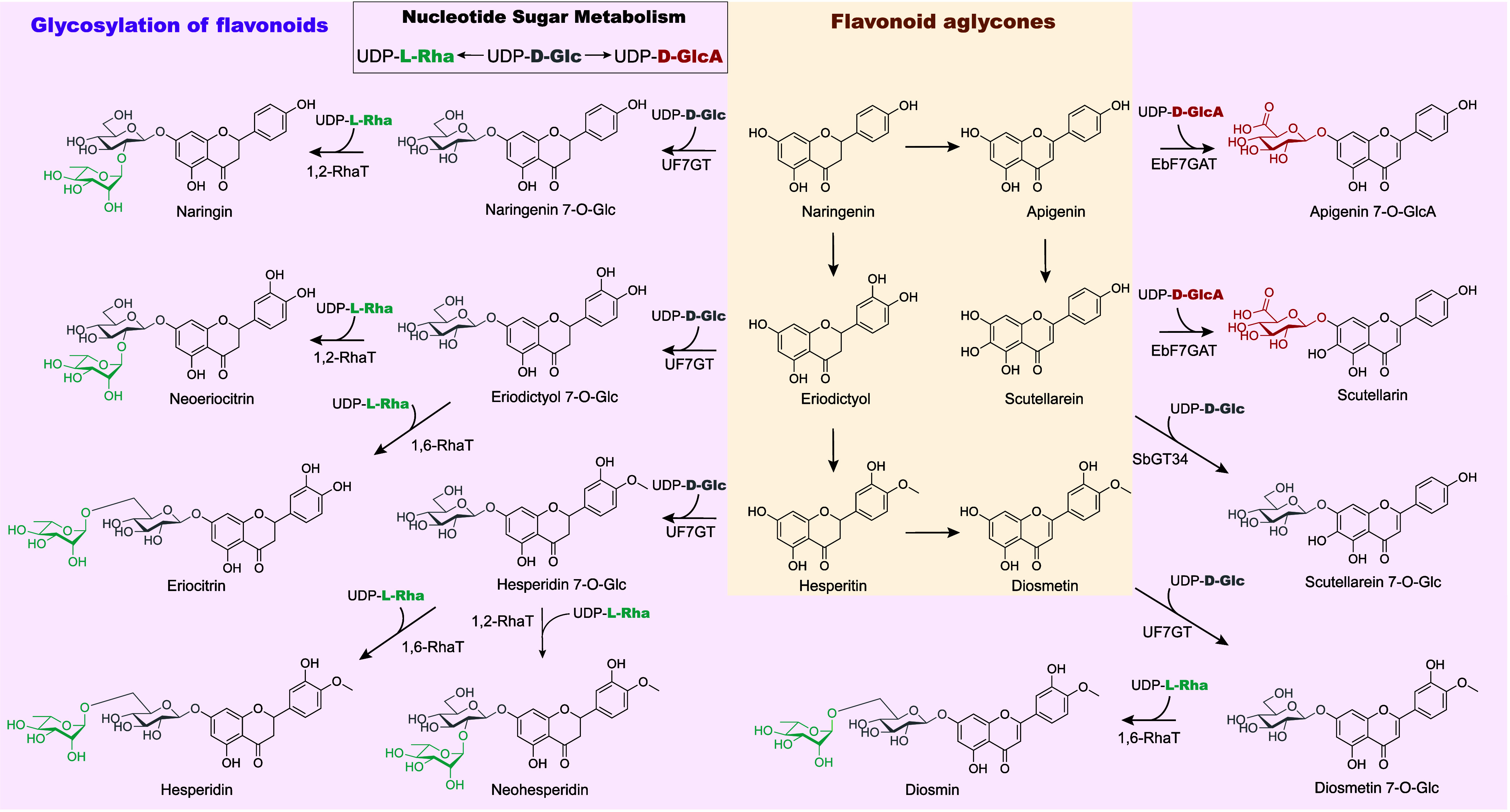
Examples of flavonoid glycosides glycosylated in *S. cerevisiae*.

Flavonoids are synthesized from phenylalanine *via* the shikimate pathway and are similarly oxidized by P450s and glycosylated by UGTs (Figure 7). The biosynthesis of flavonoids in yeast has been achieved.^48^ However, most examples of this type of glycosylation employ the feed-in of the associated flavonoid aglycone due to challenges in engineering the full pathway.

### Increasing UDP-d-Glc Pools and Knocking out Glucosidases in Improving Flavonoid-7-O-Glc Production

For the production of flavonoid-7-O-glycosides by *S. cerevisiae*, efforts have been made to engineer UDP-d-Glc pools and knock out known endogenous glucosidases. To increase production of glycosylated products, the deletion of endogenous glucosidases will be crucial, as these enzymes can noticeably degrade production.^49^ In increasing the titer of naringenin-7-O-Glc production in *S. cerevisiae*, Li *et al.* knocked out both known flavonoid-7-O glycoside glucosidases, EXG1 and SPR1, in addition to increasing UDP-d-Glc pools by overexpressing enzymes involved in UTP biosynthesis, UDP-d-Glc biosynthesis, and d-glucose uptake.^50^ This resulted in a 7.9-fold increase in the titer of their desired product (2*S*)-naringenin. A similar approach was taken in the production of scutellarein 7-O-glucoside, where UGP1 and PGM2 were overexpressed and the genes encoding the native β-glucosidases, EXG1, SPR1, and YIR007W, were knocked out, resulting in 1.2 g/L of scutellarein 7-O-glucoside produced in a 4 L reactor supplemented with ∼3.5 g of scutellarein.^51^ These classic metabolic engineering strategies of knocking out degradation enzymes and overexpressing upstream enzymes to increase precursor pools are important to improve glycoside biosynthesis. Specifically increasing UDP-d-Glc pools will be important for glycosylating with nucleotide sugars that are derived from UDP-d-Glc, such as UDP-d-GlcA, UDP-l-Rha, *etc.*, due to complicated feedback mechanisms that preserve UDP-d-Glc levels, as highlighted in the previous section of this review.

### Non-glucose Glycosylation in *S. cerevisiae*

As mentioned before, most reports of natural product glycosylation in yeast have been restricted to glucosylations, which can take advantage of the endogenous pool of UDP-d-Glc in yeast. However, there are several examples of successful glycosylations. Flavonoids that serve as precursors to breviscapine were synthesized in yeast, including the production of apigenin 7-O-GlcA and scutellarin (which contains a d-GlcA residue) by introducing EbUGD and its respective flavonoid 7-O-glucuronosyltransferase EbF7GAT from *Erigeron breviscapus*; titers were increased by increasing the flux of the precursor malonyl-CoA to improve flavonoid production (Figure 6).^13^ Recently, the biosynthesis of flavonoid 7-O-disaccharides was reported in *S. cerevisiae*, specifically the production of eriocitrin, naringin, hesperidin, neohesperidin, diosmin, and neoeriocitrin, each of which contains either a d-glucose-(1,2)-l-rhamnose or d-glucose-(1,6)-l-rhamnose disaccharide.^14^ The biosynthesis of precursor UDP-l-Rha was achieved by introducing RHM from *A. thaliana* into *S. cerevisiae*, and further optimization of glycosylation was achieved by both creating a chimeric UDP-l-Rha-producing enzyme that is able to regenerate NADH and overexpressing upstream enzymes in UDP-d-Glc biosynthesis, resulting in titers of hundreds of mg/L of flavonoid 7-O-disaccharides with a feed-in of the respective aglycones.^14^

*S. cerevisiae* has been important in the discovery of pathways in saponin synthesis and the characterization of UGTs. The production of glycyrrhizin, a desired sweetener that is 150 times sweeter than sucrose, was achieved at 225.3 μg/L in *S. cerevisiae* with the discovery of a cellulose synthase-like enzyme that glucuronidates the C3 residue of glycyrrhetinic acid and expression of UGD from *A. thaliana* to produce UDP-d-GlcA.^9^ This was reported at the same time as the discovery that cellulose synthase-like enzymes are involved in the pathway for producing yossoside V, an abundant saponin found in spinach, where the production of medicagenic acid 3-O-GlcA was achieved in *S. cerevisiae* with UGD from *Spinacia oleracea*, producing UDP-d-GlcA.^8^ However, significant amounts of unglycosylated medicagenic acid remained, showing that there is an opportunity for optimization to either improve glycosyltransferase activity or UDP-sugar availability.^8^ Yossosides I–V also contain a d-fucose residue, and since there was no known UDP-d-Fuc biosynthetic pathway, the fucosylation was tested and confirmed in *Nicotiana benthamiana*. The recent discovery of the biosynthesis of UDP-d-Fuc *via* UDP-d-Glc 4,6-dehydratase and a homologue of neomenthol dehydrogenase is exciting for the potential to produce many glycosylated products that contain this elusive sugar.^26^ The addition of d-xylose to make both notoginsenoside R1 and R2 was achieved in yeast with the integration of AtUGD and AtUXS to produce UDP-d-Xyl as well as the discovery of UGT94Q13 that performs the xylosylation to produce both saponins.^52^

There have been some efforts in engineering heterologous nucleotide sugar pathways from plants and microbes in yeast to study the metabolism of nucleotide sugar interconversion.^53^ Particularly, delaying the production of UDP-d-Xyl can allow for higher accumulation of the upstream product UDP-d-GlcA.^53^ Also, it was observed that the production of UDP-l-Rha can inhibit the production of UDP-d-Fuc.^53^ Studies such as these may become more important to understand bottlenecks caused by UDP-sugar interconversion limits.

### Engineering UGTs and Their Expression in *S. cerevisiae*

There are a few examples of increasing glycosylation efficiency either by traditional metabolic engineering methods of increasing enzyme expression through promoter engineering and testing homologues^54^ or by using site-directed mutagenesis to increase enzyme activity.^47^ Production of ginsenoside Rh2 at 2.25 g/L in a 10 L bioreactor (79.3 mg/L in shake flasks) was achieved in *S. cerevisiae* both through engineering higher titers of the aglycone protopanaxadiol by balancing P450 levels and by improving the C3 glycosylation conversion through increasing the copy number, improving protein expression through promoter engineering, and increasing enzyme activity through directed evolution.^54^ There have been some efforts to engineer UGTs that can perform a wide variety of glycosylation reactions. The production of artificial sweeteners rebaudioside D (Reb D) and rebaudioside M (Reb M) was increased in *S. cerevisiae* relative to alternative products 1,2-stevioside and rebaudioside A by mutating UGT76G1, an enzyme that is able to glucosylate both steviol C13 and C19 residues and perform up to eight different glucosylation reactions.^47^ These mutations were achieved by homology modeling to target 1,3-glycosylation and site-directed mutagenesis that was then screened to find enzyme mutants that increased accumulation of Reb D and Reb M.^47^ In order to screen large libraries of mutants and homologues of UGTs, coupled *in vitro* fluorescence assays that could be used to screen UGT variants in a 384-well format though the correlation between *in vitro* and *in vivo* in *S. cerevisiae* would need to be tested.^55^

## Outlook

It is a very exciting time to engineer the production of heterologous products in yeast with the discovery of new biosynthetic pathways and the development of new strategies for metabolic engineering. While this review has focused on small-molecule glycosylation, yeast is also a potential host for production of other glycosylated products such as proteins.^56^ It is important to note that multiplexed approaches will be necessary to balance UDP-sugar concentration, concentration of aglycones, and enzyme expression.

Beyond UGTs and precursors, other efforts also involve the engineering of yeast’s native morphology to increase glycosylated products, for example, the expansion of the endoplasmic reticulum to allow for a larger area for P450 docking to increase product titers. The knockout of phosphatidic acid phosphatase (PAH1), an enzyme that generates neutral triglycerides from phosphatidic acid in *S. cerevisiae*, increased the production of medicagenic acid C28-O-Glc 16-fold.^57^ The expansion of the ER can be achieved by overexpressing INO2, an important ER size regulatory factor, that increased production of the ginsenoside aglycone protopanaxadiol 8-fold in *S. cerevisiae*.^58^ The engineering of yeast to produce such complexly decorated molecules will continue to require a careful balance of relative gene expression of a variety of enzymes, production balancing of various precursors, and metabolic burden.

With the synthesis of large molecules that come from heterologous species, transporters and accessory proteins may become increasingly important for the glycosylation of these natural products. The plant vacuole is often used to store secondary metabolites like saponins, and ABC-type transporters are likely used to allow these metabolites to enter the vacuole.^59,60^ For example, avenacin A1 is a saponin found in *Avena strigosa,* where the last two biosynthetic steps occur in the vacuole, including the final glycosylation step, and this molecule could be synthesized in *N. benthamiana* without the addition of any transporters.^15,61^ These transporters are largely uncharacterized, though it is likely that many plants share this machinery. Recent achievements in the production of medicinal tropane alkaloids in *S. cerevisiae* show that it is possible to engineer up to six subcellular compartments in yeast, including the vacuole and the peroxisome, for the production of heterologous small products.^6^

It may also be advantageous to utilize yeast native compartments, such as the peroxisome, for heterologous natural product biosynthesis. The peroxisome has already been repurposed from its native function to easily import heterologously expressed enzymes to produce natural products, showing its potential as a compartment of engineered specialized metabolism.^62^ It has also been used for the storage of protopanaxadiol in the peroxisomal membrane to increase production by 78%.^63^

As stated in the Introduction, *S. cerevisiae* is an advantageous platform compared with others for a variety of reasons. Yeast may also express heterologous enzymes like nucleotide sugar enzymes found in plants, whereas other microbes like *Escherichia coli* have a hard time expressing activated forms of the enzyme UGD that produces UDP-d-GlcA.^64,65^ Yeast also grows much faster compared to native producers of these products, such as plants, which can take decades to mature. Alternative plant platforms such as *N. benthamiana* offers the advantage of containing more metabolic precursors, such as nucleotide sugar diversity, and possibly contain other helper enzymes not yet identified.^2^ However, the most sufficient of these methods requires transient expression using agrobacterium infiltration that produces gram-scale quantities on the scale of months. We hope that the expansion of nucleotide sugar metabolism in yeast will lead to the establishment of stable strains that can easily be used to test different glycosylations in a matter of weeks and eventually establish stable strains for the large-scale production of glycosylated molecules.

There are significant challenges with balancing the production of UDP-sugar metabolism with internal feedback mechanisms. With the elucidation and engineering of UDP-sugar metabolism and UGTs, not only will there be more production of glycosylated natural and new-to-nature products and proteins but also a greater understanding of glycosylation in general.

## Supporting Information Available

Supporting table for Figure 2 including list of enzymes and example origin (PDF)
