# Supplementary material for: Advances in Engineering Nucleotide Sugar Metabolism for Natural Product Glycosylation in Saccharomyces cerevisiae
Source: ACS Synth Biol. 2024 May 31;13(6):1589–99. doi: 10.1021/acssynbio.3c00737 (PMC11197093; doi:10.1021/acssynbio.3c00737)
Supplement: Supplementary file 1 — sb3c00737_si_001.pdf [file sb3c00737_si_001.pdf]

Supporting information for

Advances in Engineering Nucleotide Sugar Metabolism for Natural Product Glycosylation in *Saccharomyces cerevisiae*

Samantha A Crowe[1,2,3], Yuzhong Liu[2,3], Xixi Zhao[2,3], Henrik V. Scheller[3,4,5], Jay D. Keasling[1,2,3,6,7,8,9*]

[1] Department of Chemical & Biomolecular Engineering, University of California, Berkeley, CA, USA
[2] California Institute of Quantitative Biosciences (QB3), University of California, Berkeley, CA, USA
[3] Joint BioEnergy Institute, Emeryville, CA, USA
[4] Environmental Genomics and Systems Biology Division, Lawrence Berkeley National Laboratory, Berkeley, CA, USA
[5] Department of Plant and Microbial Biology, University of California, Berkeley, CA, USA
[6] Department of Bioengineering, University of California, Berkeley, CA, USA
[7] Division of Biological Systems and Engineering, Lawrence Berkeley National Laboratory, Berkeley, CA, USA
[8] Center for Biosustainability, Danish Technical University, Lyngby, DK
[9] Center for Synthetic Biochemistry, Shenzhen Institutes for Advanced Technologies, Shenzhen, China

*Correspondence: keasling@berkeley.edu (J.D.K.)

Table of Contents

Table S1. List of enzymes and example origin in Figure 2..S3

Table S1. List of enzymes and example origin in Figure 2.

Substrate(s)	Product(s)	Enzyme Name	Enzyme Abbrev.	Note
Sucrose + H_2O	D-Fru + D-Glc	Invertase/hydrolases	Suc2p, etc	*S. cerevisiae* native metabolism[1]
D-Fru + ATP	D-Fru-6-P + ADP	Fructokinase	FruK	*S. cerevisiae* native metabolism[2]
D-Glc + ATP	D-Glc-6-P + ADP	Glucokinase	GlcK	*S. cerevisiae* native metabolism[3]
D-Glc-6-P	D-Fru-6-P	D-Glc-6 isomerase	GPI	*S. cerevisiae* native metabolism[4]
D-Fru-6-P + Gln	D-GlcN-6-P + D-Glc	D-GlcN-6-P Synthase	GFA1	*S. cerevisiae* native metabolism[5]
D-GlcN-6-P +AcCoA	D-GlcNAc-1-P + CoA	D-GlcN-6-P acetyltransferase	GNA1	*S. cerevisiae* native metabolism [5]
D-GlcNAc + UTP	UDP-D-GlcNAc + PP_i	UDP-GlcNAc pyrophosphorylase	UAP1/QRI1	*S. cerevisiae* native metabolism[5]
UDP-D-GlcNAc	UDP-D-GalNAc	UDP-Gal/GalNAc-4-epimerase	UGE	*S. cerevisiae* native metabolism[5]
D-Glc-6-P	L-inositol-1-P	L-inositol 1-P synthetase	IPS	*S. cerevisiae* native metabolism[6]
L-inositol-1-P + H_2O	Inositol + P_i	Inositol monophosphatase	IMP	*S. cerevisiae* native metabolism[7]
Inositol + O_2	D-GlcA + H_2O	Myo-inositol oxygenase	MIOX	Can be found in plants[8]
D-Glc-6-P	D-Glc-1-P	Phosphoglucomutase	PGM	*S. cerevisiae* native metabolism[9]
D-Glc-1-P + UTP	UDP-D-Glc + PP_i	UDP-D-Glc pyrophosphorylase	UGP1	*S. cerevisiae* native metabolism[9]
UDP-D-Glc	UDP-D-Gal	UDP-D-Glc 4-epimerase	UGE	*S. cerevisiae* native metabolism[9]
D-Gal + ATP	D-Gal-1-P + ADP	Galactokinase	GalK	*S. cerevisiae* native metabolism[9]
D-Gal-1-P + UD-D-Glc	UDP-D-Gal + D-Glc-1-P	Galactose-1-phosphate uridylyltransferase	GALT	*S. cerevisiae* native metabolism[9]
Sucrose + UDP	UDP-D-Glc + D-Fru	Sucrose synthase	SuSy	Can be found in plants[10]
UDP-D-Gal	UDP-D-Gal*f*	UDP-D-Gal mutase	UGM	Can be found in bacteria, fungus and plants[11]

UDP-D-Glc + 2NAD^+ + H_2O	UDP-D-GlcA + 2NADH	UDP-D-Glc 6-dehydrogenase	UGD	Can be found in plants[10]
UDP-D-GlcA	UDP-D-GalA	UDP-D-GlcA 4-epimerase	UGlcAE	Can be found in plants[10]
UDP-D-GlcA	UDP-D-Xyl + CO_2	UDP-D-Xyl Synthase	UXS	Can be found in plants[10]
UDP-D-GlcA	UDP-D-Api/UDP-D-Xyl + CO_2	UDP-D-Api/Xyl Synthase	AXS	Can be found in plants[10]
UDP-D-Xyl	UDP-L-Ara*p*	UDP-Xylose 4-epimerase	UXE	Can be found in plants[10]
UDP-L-Ara*p*	UDP-L-Ara*f*	UDP-Ara mutase	UAM	Can be found in plants[10]
UDP-D-Glc + NAD^+ + NADPH	UDP-L-Rha + NADH + $NADP^+$ + H_2O	UDP-L-Rha synthase	RHM	Can be found in plants[10]
UDP-D-Glc + NAD^+	UDP-4-keto-6-deoxcy-D-Glc + NADH + H_2O	UDP-D-Glc 4,6-dehydratase	UG46DH	Can be found in plants, *Saponaria vaccaria*[12]
UDP-4-keto-6-deoxy-D-Glc + NADPH	UDP-D-Fuc + $NADP^+$	4-keto reductase	NMD	Can be found in plants, *Saponaria vaccaria*[12]
D-GlcA + ATP	D-GlcA-1-P + ADP	Glucuronokinase	GlcAK	Can be found in plants[10]
D-GlcA-1-P + UTP	UDP-D-GlcA + PP_i	UDP-sugar pyrophosphorylase	USP	Can be found in plants[10]
D-GalA + ATP	D-GalA-1-P + ADP	GalA-1-P kinase	GalAK	Can be found in plants[10]
D-GalA-1-P + UTP	UDP-D-GalA + PP_i	UDP-sugar pyrophosphorylase	USP	Can be found in plants[10]
L-Rha + ATP	L-Rha-1-P + ADP	Rhamnulokinase	RhaK	Can be found in plants[10]
L-Rha-1-P + UTP	UDP-L-Rha + PP_i	UDP-sugar pyrophosphorylase	USP	Can be found in plants[10]
L-Ara + ATP	L-Ara*p*-1-P + ADP	Arabinokinase	AraK	Can be found in plants[10]
L-Ara*p*-1-P + UTP	UDP-L-Ara*p* + PP_i	UDP-sugar pyrophosphorylase	USP	Can be found in plants[10]

References

(1) Marques, W. L.; Raghavendran, V.; Stambuk, B. U.; Gombert, A. K. Sucrose and Saccharomyces Cerevisiae: A Relationship Most Sweet. *FEMS Yeast Res.* **2016**, *16* (1), fov107.

(2) Lobo, Z.; Maitra, P. K. Phosphofructokinase Mutants of Yeast. Biochemistry and Genetics. *J. Biol. Chem.* **1983**, *258* (3), 1444–1449.
(3) Maitra, P. K.; Lobo, Z. Genetics of Yeast Glucokinase. *Genetics* **1983**, *105* (3), 501–515.
(4) Maitra, P. K.; Lobo, Z. Genetic Studies with a Phosphoglucose Isomerase Mutant of Saccharomyces Cerevisiae. *Mol. Gen. Genet.* **1977**, *156* (1), 55–60.
(5) Milewski, S.; Gabriel, I.; Olchowy, J. Enzymes of UDP-GlcNAc Biosynthesis in Yeast. *Yeast* **2006**, *23* (1), 1–14.
(6) Hirsch, J. P.; Henry, S. A. Expression of the Saccharomyces Cerevisiae Inositol-1-Phosphate Synthase (INO1) Gene Is Regulated by Factors That Affect Phospholipid Synthesis. *Mol. Cell. Biol.* **1986**, *6* (10), 3320–3328.
(7) Murray, M.; Greenberg, M. L. Regulation of Inositol Monophosphatase in Saccharomyces Cerevisiae. *Mol. Microbiol.* **1997**, *25* (3), 541–546.
(8) Endres, S.; Tenhaken, R. Down-Regulation of the Myo-Inositol Oxygenase Gene Family Has No Effect on Cell Wall Composition in Arabidopsis. *Planta* **2011**, *234* (1), 157–169.
(9) Daran, J. M.; Dallies, N.; Thines-Sempoux, D.; Paquet, V.; François, J. Genetic and Biochemical Characterization of the UGP1 Gene Encoding the UDP-Glucose Pyrophosphorylase from Saccharomyces Cerevisiae. *Eur. J. Biochem.* **1995**, *233* (2), 520–530.
(10) Bar-Peled, M.; O’Neill, M. A. Plant Nucleotide Sugar Formation, Interconversion, and Salvage by Sugar Recycling. *Annu. Rev. Plant Biol.* **2011**, *62*, 127–155.
(11) Seničar, M.; Lafite, P.; Eliseeva, S. V.; Petoud, S.; Landemarre, L.; Daniellou, R. Galactofuranose-Related Enzymes: Challenges and Hopes. *Int. J. Mol. Sci.* **2020**, *21* (10). https://doi.org/10.3390/ijms21103465.
(12) Chen, X.; Hudson, G. A.; Mineo, C.; Amer, B.; Baidoo, E. E. K.; Crowe, S. A.; Liu, Y.; Keasling, J. D.; Scheller, H. V. Deciphering Triterpenoid Saponin Biosynthesis by Leveraging Transcriptome Response to Methyl Jasmonate Elicitation in Saponaria Vaccaria. *Nat. Commun.* **2023**, *14* (1), 7101.
